# Individual differences in temperaments traits and complex network properties of fMRI

**DOI:** 10.1186/1471-2202-13-S1-P114

**Published:** 2012-07-16

**Authors:** Sunghyon Kyeong, Won Sup Kim, Dong-Uk Hwang

**Affiliations:** 1 Division of Computational Sciences in Mathematics, National Institute for Mathematical Sciences, Daejeon, Republic of Korea

## 

In the previous study, Cloninger’s psychobiological model of personality [1,2] revealed that temperament traits are genetically determined. Thus, it is postulated that temperament traits determine one’s disposition to the early emotions of fear, anger and attachment and one’s automatic behavioral responses to the environmental stimuli of danger, novelty and reward. The previous studies have revealed the correlational tendency between the brain volume size and temperament traits [3,4]. However, only a few studies reported that neural correlates of the temperament traits in the resting state functional network. Thus, it is required to analyze function network and its neural correlates of temperament traits to advance our understanding on how psychobiological properties are correlated to functional network.

Forty young male volunteers (mean age=25.2±3.3 years) took part in this experiment. None of these participants had a history of traumatic brain injury, epilepsy, or other neurological and psychiatric problems. The present study was approved by the Institutional Review Board of Severance Hospital, and all volunteers gave informed consent before participating this experiment. The Korean version of the temperament and character inventory (TCI) was used to assess temperament and character factors of all participants [5]. The participants underwent the resting state fMRI scanning (404 volumes with TR=2000ms). The participants were instructed to keep resting state with eyes closed. After applying the spatiotemporal preprocessing, we divided the whole brain regions into 1000 sub-regions by using the previously reported methods [6] and obtained the mean time series for each sub-region. The adjacency matrix was investigated by using the Pearson’s correlation coefficients between all pairs of mean time series. We measured network properties and performed group comparisons of the network measures.

The *k*-means clustering algorithm with three temperament personality traits (i.e. NS, HA, and RD scores) of 40 subjects separates the sample data into two groups. 18 (22) subjects are classified as obsessional (histrionic) personality group. The correlation analysis of temperament traits and the complex network measures show that the node strength in right middle frontal, right supplementary motor areas has decreased in OP. However node strength in right cerebellum has increased in OP.

## Conclusion

Our study was the first attempt to distinguish personality disorder subtypes by using *k*-means clustering algorithm with three temperament traits. Based upon the clustering results, we performed statistical comparison on network measures between histrionic and obsessional personality groups.
